# Endoscopic surgery suturing techniques: a randomized study on learning

**DOI:** 10.1186/s12893-022-01513-2

**Published:** 2022-02-17

**Authors:** F. J. Voskens, E. M. van der Schans, J. P. Ruurda, I. A. M. J. Broeders

**Affiliations:** 1 Department of Surgery, Meander Medical Center, Maatweg 3, Amersfoort, The Netherlands; 2 Department of Gastro-Intestinal and Oncologic Surgery, University Medical Center, Utrecht, The Netherlands; 3 University of Twente, Robotics and Mechatronics, Enschede, The Netherlands

**Keywords:** Surgical robotics, Laparoscopic suturing, Robotic suturing, Learning curve, Medical robots and systems

## Abstract

**Background:**

Surgeons have widely adopted endoscopic suturing techniques using conventional laparoscopic instruments and the more advanced robotic systems. The FlexDex is a novel articulating laparoscopic needle driver providing enhanced dexterity in laparoscopic surgery. This study evaluates and compares the learning curve of endoscopic suturing with conventional laparoscopy, the FlexDex and robotic suturing in novices.

**Methods:**

Participants performed a minimal invasive suturing task in three different ways in a randomized order: with a conventional laparoscopic needle driver, using the FlexDex needle driver and third, using the Da Vinci Si surgical system. Primary outcome was suturing task time. Secondary outcome parameters were assessment of suturing quality and workload perception.

**Results:**

A total of 10 novice participants were included and completed a total of 300 sessions. Median (IQR) suturing time of the first 5 sessions was 231 s (188–291) in the laparoscopic group versus 378 s (282–471) in the FlexDex group versus 189 s (160–247) in the DaVinci Si group. The last 5 sessions showed significant reduction of median suturing time of 143 s (120–190), 232 s (180–265) and 172 s (134–199) respectively. Analysis identified that the learning curve for the laparoscopic needle driver and Da Vinci Si was reached in 5 sessions, compared to 8 sessions for the Flexdex. The laparoscopic needle driver and Da Vinci Si showed a significant shorter median suturing time compared to the FlexDex (p = 0.00). The FlexDex quality assessment scores were significantly lower compared to the laparoscopic (p = 0.00) and robotic (p = 0.00) scores and perceived workload remains high for the FlexDex users.

**Conclusions:**

Ex vivo endoscopic suturing with the FlexDex demonstrated a prolonged learning curve compared to laparoscopic and robotic suturing. The learning curve of the FlexDex is fundamentally different from conventional laparoscopic and robotic instruments. This study provides further insights in the implementation and training of endoscopic suturing techniques.

## Background

Over the years constant innovation and technological advancement have facilitated surgeons to expand their practice of laparoscopic surgery [1–8]. Nonetheless, laparoscopic intracorporeal suturing remains an important technical barrier in laparoscopic surgery [9, 10]. It is an advanced skill to master with an extensive learning curve, mainly due to the limited degrees of freedom of the surgical instruments and the fulcrum effect [11, 12]. The implementation of robotic systems, including the da Vinci Surgical System, offers a solution for the challenges of intracorporeal suturing [13, 14]. The 3D imaging, tremor elimination, enhanced ergonomics and articulating instruments improve surgical performance and make complex tasks such as suturing easier to perform [15, 16]. Robotic assisted suturing has demonstrated to have a shorter learning curve compared to laparoscopy and is especially beneficial when suturing in deep and confined operating space [17].

**Fig. 1:**
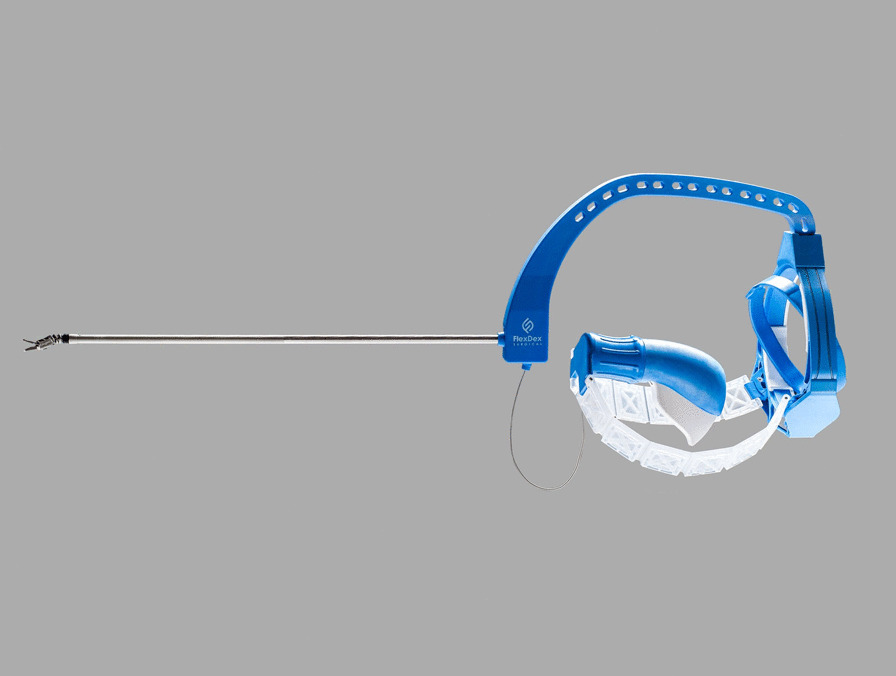
The FlexDex surgical laparoscopic needle driver

Meanwhile the majority of surgeons worldwide still do not have daily access to a robotic system [18]. The significant costs of robotic surgery remain an important restrictive factor. This financial gap between laparoscopic and robotic instruments has motivated the development of hand held articulating laparoscopic suturing instruments, such as the FlexDex Surgical laparoscopic needle driver (FlexDex, Inc., Brighton, MI), Kymerax (Terumo, Japan) [19] and the Radius Surgical System (Tuebingen Scientific. Medical GmbH, Germany)[20, 21]. These instruments provide the surgeon with some of the dexterity benefits of robotic surgery. To date the acceptance and extensive use of articulating laparoscopic instruments remains limited. One of the main reasons is the significant learning curve [22]. In order to succeed an instrument should feel as a natural extension of the arm, wrist and hand and should be easy to use. The FlexDex is a novel mechanically articulated suturing device (Fig. 1). This arm mounted device is fundamentally different from previous articulating needle holders, since it converts wrist movements into articulation of the tip of the instrument. This design allows parallel mapping, translating the movement of the surgeon into respective movement of the end effector [23]. Use of the FlexDex could improve the ergonomics of the surgeon in comparison with conventional laparoscopic instrumentation.

The extent of the learning curve of the FlexDex is unknown. It is necessary to take learning curves of innovative instruments into account since it puts the effectiveness of innovations into perspective. The aim of this study was to evaluate and compare the learning curve of minimal invasive suturing by novices using the FlexDex, conventional laparoscopic needle driver and the Da Vinci Si surgical system. Our hypothesis was that the learning curve of the FlexDex is longer compared to laparoscopic and robotic suturing, since the device seems more complex compared to laparoscopic and robotic suturing. We expect novices to have the shortest learning curve of minimal invasive suturing with the DaVinci Si, due to the benefits in interface, dexterity and ergonomics [24].

## Methods

### Study setting

This randomized crossover study was performed at the Meander Medical Center Amersfoort, The Netherlands. Ten junior residents without experience in laparoscopic surgery were recruited. Residents with prior endoscopic suturing experience were excluded from participation. Baseline demographic data of the participants were collected at the time of consent and participation was voluntary. Participants were consecutively randomized into one of the 3 groups by means of sealed envelope technique to determine the order of device of each session (e.g., ABC, BCA, CAB for 3 devices A, B and C). The first group started using the conventional laparoscopic needle driver (A), followed by the Da Vinci Si (B) and the FlexDex (C). The second group started using the Da Vinci Si and the third group started with the FlexDex. After completion of the first session the sequence per group alternated.

### Suturing task

The suturing tasks were performed within a laparoscopic box trainer environment (d-box laparoscopic simulator). The participants started the study with a brief training of 10 min on each modality and every session started with a ‘rope passing’ exercise designed for familiarization. This exercise was not part of the evaluation. Subsequently the participant performed the suturing task. A suturing pad with three premarked targets as exit points at 1-cm intervals was placed with an angle of 45° to the scope. The first step consisted of passing the needle through the most cranial target. This was followed by tying one surgeon’s knot and two square ties. The task was finished by passing the needle through the two remaining targets. Trocar placement, camera position, angle of the laparoscope and suture material (Vicryl 3–0 SH plus 26 mm, Ethicon, Johnson & Johnson) were standardized. Laparoscopic imaging was in 3D, in order to limit difference to the da Vinci Si surgical system imaging. In this study we used the 10 mm 3D EndoEye Star videoscope of Olympus for 3D laparoscopy. The tasks were completed bimanually. A reusable laparoscopic grasper was used as the left instrument during suturing with the conventional laparoscopic needle driver and the FlexDex. Two arms, next to the camera arm, were docked on the da Vinci Si system: the Wristed Needle Driver on the right side and the Cadiére Forceps on the left side. A maximum of 600 s was given to complete the suturing task per device. If suture breakage occurred, the participant started over and the time of the previous broken suture was added. Previous studies have shown that the learning curve for both laparoscopic and robotic instrumentation improved within five sessions and is flattened by the 10th session [17, 25]. Since the learning curve in the current study was expected to be comparable, each participant performed 10 session with each suturing device.

### Outcome variables

The primary outcome of this study was task efficiency, defined as suturing task time (s). Secondary outcomes were the quality assessment of the laparoscopic suturing skills and the workload assessments. The suturing task was recorded by a digital video recorder and coded to blind the study session for the observers. Analysis of the videos was performed by 2 blinded observers with the Global Operative Assessment of Laparoscopic Skills (GOALS) checklist. This is a 29-point validated checklist for the assessment of endoscopic suturing skills [26]. Following each suturing task the participants were asked to complete the National Aeronautics and Space Administration Task Load Index (NASA-TLX) [27]. The NASA-TLX is a validated workload assessment tool, measuring self-ratings of satisfaction, performance and fatigue of the participants.

### Statistical analyses

Data analysis was performed in consultation with a statistician. Continuous variables were assessed for normality and were presented as medians and interquartile ranges if not normally distributed. Nonparametric tests were used for statistical analyses. The Kruskal Wallis test was used to analyze the differences in outcomes between the laparoscopic, robotic and FlexDex group. If the groups differed significantly, pairwise comparisons were performed to determine the significance between each group using the Mann–Whitney U test. The Wilcoxon signed rank test was used to test improvement within each group, comparing the first five sessions and last five sessions. Data was analyzed using SPSS version 26, with the significance level set at p = 0.05.

## Results

**Fig. 2:**
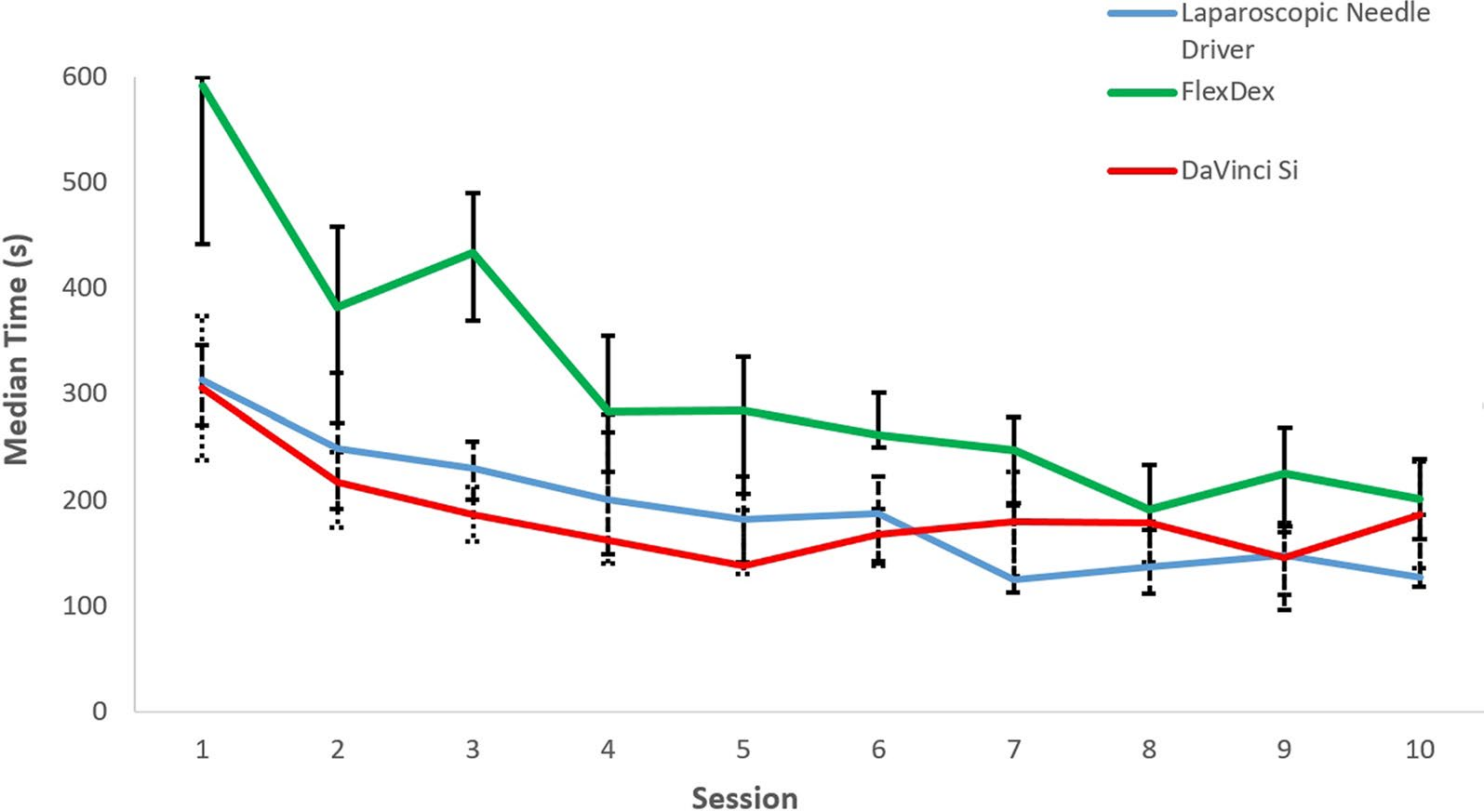
Median time with IQR suturing task

Mean age of the ten participants was 30 years and the male–female ratio was 8:2. Together, the participants completed a total of 300 sessions. Suture breakage occurred in eight sessions, all with the DaVinci Si. The median completion time of the suturing task is displayed in Fig. 2. Visual analysis of the curve of the conventional laparoscopic and robotic group shows the same pattern of improvement with a plateau reached after 5 sessions. The most rapid decline is seen in the FlexDex group. Median completion time at the start in the FlexDex group is high and a plateau in time is reached after 8 sessions. 5 participants could not complete the suturing task with the FlexDex at their first sessions within the time allotted (600 s). The resulting median times per suturing device are shown in Table 1, set out in the first and last five sessions. Median (IQR) suturing time of the first 5 sessions was 231 s (188–291) in the laparoscopic group versus 378 s (282–471) in the FlexDex group versus 189 s (160–247) in the DaVinci Si group. Analysis of the last 5 sessions showed significant reduction of median suturing time of 143 s (120–190), 232 s (180–265) and 172 s (134–199) respectively. Minimal invasive suturing with the FlexDex remained significantly slower throughout the study compared with laparoscopic and robotic assistance (p = 0.00).

**Table 1:** Total median time (s) of the suturing task per device

	Laparoscopic needle driver (n = 100)	FlexDex (n = 100)	DaVinci Si (n = 100)	p value^(a)^	Post hoc analysis^(b)^		
					Lap vs FlexDex	FlexDex vs DaVinci Si	Lap vs Da Vinci Si
Session 1–5	231 s (188, 291)	378 s (282, 471)	189 s (160, 247)	0.00	0.00	0.00	0.05
Session 6–10	143 s (120, 190)	232 s (180, 265)	172 s (134, 199)	0.00	0.00	0.00	0.07
p value^(c)^	0.00	0.00	0.00				

Values are expressed in median and interquartile range

n = total number of sessions

^(a)^Kruskal Wallis Test

^(b)^Mann–Whitney U test

^(c)^Wilcoxon signed rank test

**Fig. 3:**
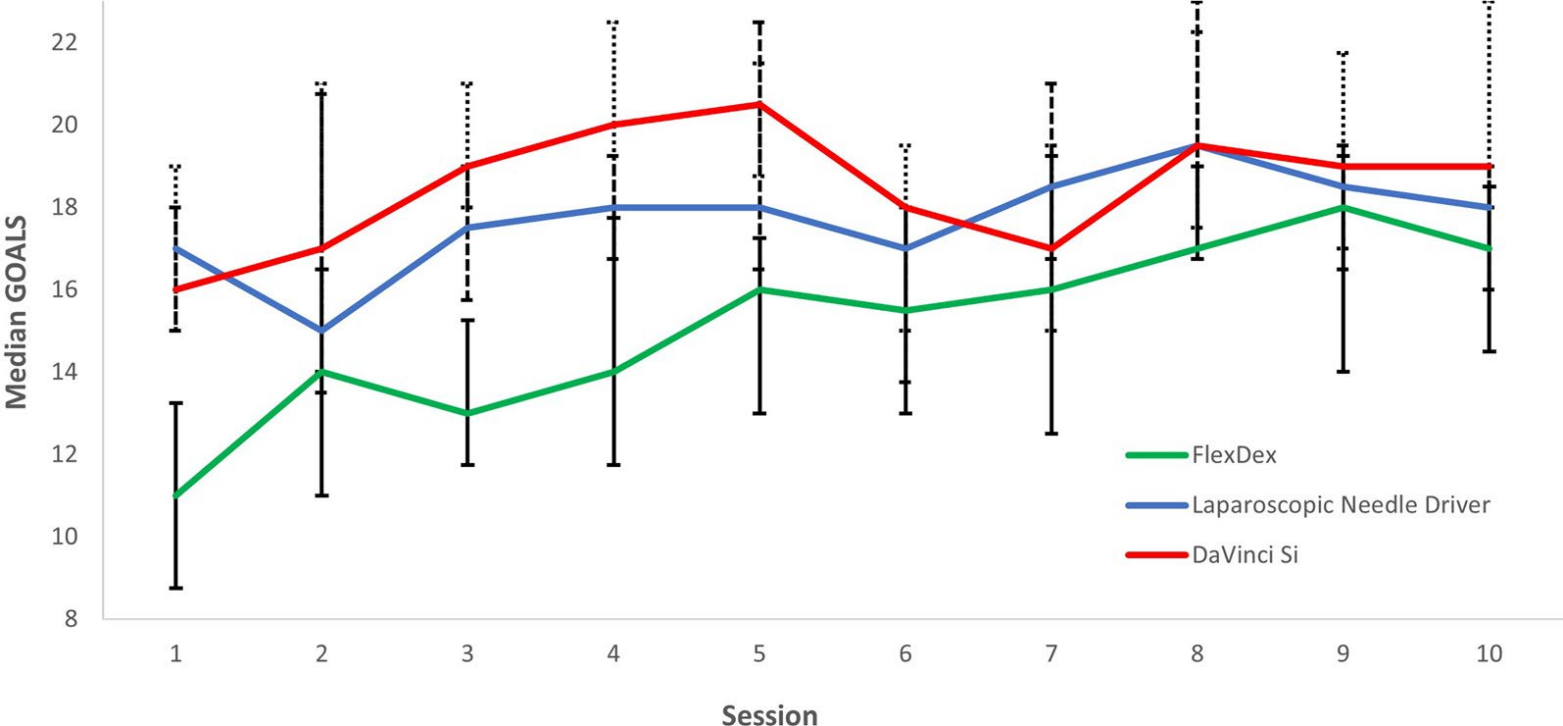
Median checklist scores (GOALS) of the suturing videos

**Fig. 4:**
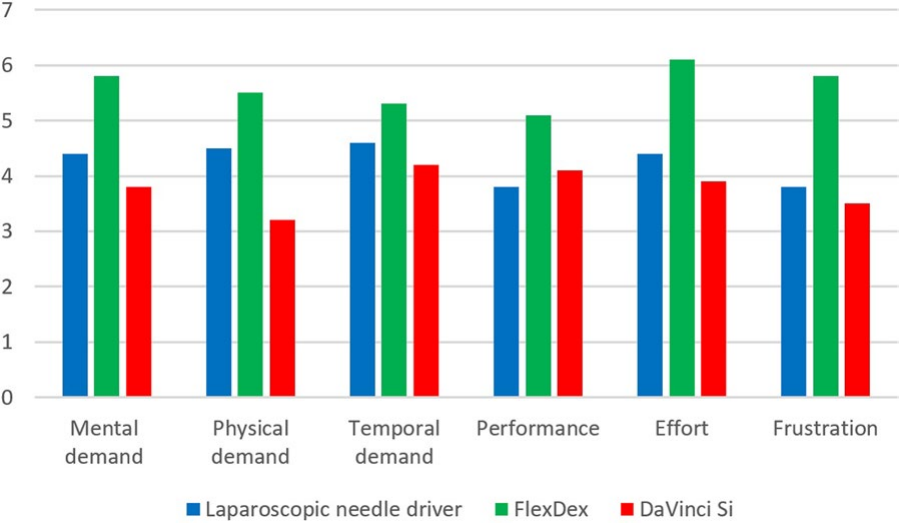
Mean workload items of NASA-TLX scores

Table 2 and Fig. 3 present the performance scores of the GOALS checklist. Overall the FlexDex scored significantly lower when compared to the laparoscopic needle driver and the Da Vinci Si. There were no significant differences between the GOALS scores of the laparoscopic needle driver and Da Vinci Si. Direct comparison of the first 5 sessions with the last 5 sessions showed statistically significant improvement for the FlexDex (p < 0.00). The DaVinci Si and laparoscopic needle driver did not show significant improvement in GOALS scores between the first and last five sessions. The mean workload scores for each of the six subscales of the NASA-TLX tool are presented in Fig. 4. Analysis of the six subscale scores demonstrated that the perceived workload with the FlexDex were highest on each of the subscales.

**Table 2:** Total median checklist scores (GOALS) of the suturing videos

	Laparoscopic needle driver	FlexDex	DaVinci Si	p value^(a)^	Post hoc analysis^(b)^		
					Lap vs FlexDex	FlexDex vs DaVinci Si	Lap vs Da Vinci Si
Session 1–5	17.5 (15.3, 19)	13 (11, 16)	19 (16, 21)	0.00	0.00	0.00	0.17
Session 6–10	18 (16.8, 19.3)	17 (14.5, 19)	18 (16, 20)	0.01	0.01	0.00	0.77
p value^(c)^	0.29	0.00	0.81				

Values are expressed in median and interquartile range. A higher score indicates a better result, maximum score of 29

^(a)^Kruskal Wallis Test

^(b)^Mann–Whitney U test

^(c)^Wilcoxon signed rank test

## Discussion

This randomized, cross-sectional study confirmed a prolonged learning phase of endoscopic suturing with the FlexDex for novices compared to laparoscopic and robotic suturing. The FlexDex learning curve has a significantly high median suturing time at the start and is followed by a steep learning curve. Endoscopic suturing with the laparoscopic needle driver and the DaVinci Si showed similar characteristics in learning curve peaks, however the robotic group showed shorter median suturing time in the first five sessions. This study indicates that manipulation of the FlexDex is different from conventional laparoscopic and robotic systems and requires more training.

Limited research has been done on mechanically articulated suturing devices [21, 28, 29]. The Radius Surgical System is the only commercially available mechanically articulated suturing device of which learning curve studies have been published. The Radius Surgical System has a different design compared to the FlexDex, flexion is controlled by deflecting the instrument handle and rotation is controlled by a thumb knob on the handle. One study has evaluated the learning curve of one single surgeon with the Radius in performing intracorporeal urethra-vesical anastomosis on a pelvitrainer and stated a learning curve of less than 10 anastomoses [28]. Results from another study of one surgeon using the Radius on a colorectal anastomosis showed different results. They suggested that proficiency was achieved after 21 procedures, following a steep learning curve [28]. Despite the limited number of participants and the varying study results, these studies provide insight in the unique learning curve of articulating laparoscopic instruments: fast improvement after a challenging slow start. In the present study the completion time with the FlexDex was high in the first five sessions, mostly accounted by the fact that 5 participants could not complete the suturing task at their first session within the time allotted (600 s). This is however followed by a rapid decline in completion time. We could conclude that it needs more time to allow novice users to convert the rotational movements of the wrist and arm with the Flexdex into precise movements at the instrument tip.

Different parameters exist to demonstrate a learning process [17, 30]. In addition to task completion time analysis, the performance curve of laparoscopic and robotic suturing showed a consistently high performance with limited increase during the study. In contrast, the median performance scores of the FlexDex were significantly lower (p < 0.05) than laparoscopic and robotic suturing and showed steady improvement throughout the study. Moreover, it did not reach a plateau phase at the end of the study. This suggests persistent learning of the participants and indicates the inability for FlexDex users to deliver the same suturing quality as seen in the robotic and laparoscopic group at this stage of their learning curve. The importance of incorporating multiple assessment parameters in a learning curve study was previously demonstrated [24]. This study compared the learning curve of laparoscopic versus robot assisted suturing on two different simulators. Outcome parameters were median time, off screen percentage and adequate knot percentage which resulted in three different learning curves. Median completion time and off screen percentage favoured the robot assisted group and adequate knot percentage favoured the laparoscopic group.

This study was performed on novice users without minimal invasive suturing experience. In future studies it will be interesting to study the learning curve for experienced surgeons [24]. Surgeons already mastering minimal invasive suturing skills could acquire proficiency of a new device more easily, since they do not have to concentrate on the technical aspects of suturing and knot tying. Furthermore, translation to the clinical operative setting would be of value to identify the additive value of the FlexDex in complex surgery. The ergonomics and effectiveness of the FlexDex has been evaluated in small clinical studies [23, 29]. Improved ergonomics and effectiveness of the FlexDex at difficult anatomic locations was demonstrated. The current study showed high workload scores for the FlexDex users. In this study these ergonomic benefits were not reflected in the reported workload scores. This is probably caused by the prolonged learning process of the participants.

We can support the assumption that surgeons, after adequate training with the FlexDex, could benefit from the increased dexterity and the preservation of haptic feedback. Currently the Da Vinci surgical system does not have haptic feedback capabilities. Haptic feedback offers useful information about the tension of a knot during suturing. Our study supports the importance of haptic feedback for novices, since suturing with the DaVinci Si resulted in a relatively high amount of suture breakage (n = 8), a problem that has been described in previous studies [31, 32]. In this study all suture breakages occurred during knot tying. Novice participants have difficulties to assess the applied forces of the robotic instruments on the suture. In practice, surgeons need to compensate the lack of haptic feedback by visual clues and experience [33].

The main strengths of this study are the study design and the inclusion of three different suturing modalities. Another strength is the use of a box trainer with real instruments. The majority of previous learning curve studies on endoscopic suturing used virtual reality simulators with different simulator modalities and outcome parameters, limiting direct comparison and resulting in bias [17, 24].

This study has several limitations to be mentioned. First is the limited number of endoscopic suturing sessions. Although we clearly denoted a prolonged learning curve in FlexDex users, there was still persistent learning visible for suturing quality in the FlexDex group.

The second limitation of our study involves the intracorporeal suturing task. A more challenging suturing task within a limited working space might have demonstrated the articulating benefits of the FlexDex and the Da Vinci Si more substantial compared to conventional laparoscopy. Another limitation is the unbalanced group size in the block randomization, resulting in a total of 34 sequences starting with laparoscopy and 33 sessions starting with the FlexDex or the Da Vinci Si. There is a possibility of bias, however this design was accepted due to logistics. The minor imbalance in device sequence is not expected to have a significant effect on the results.

Notwithstanding these limitations, this study offers insight into the learning curve of the FlexDex in relation to other minimal invasive suturing modalities. For now, we consider surgeons most proficient at acquiring profit of the FlexDex are those with high exposure in intracorporeal suturing, such as in bariatric surgery. Furthermore, it could be very interesting to use the FlexDex in procedures with limited working space, such as in transanal minimally invasive surgery and paediatric surgery. Nonetheless this study indicates that surgeons need proper training and should not use the FlexDex for that one stitch a year.

## Conclusions

This is the first study to report on the learning curve of the FlexDex, a novel mechanically articulating needle driver. The use of the FlexDex demonstrated a longer but steeper learning curve during minimal invasive suturing in novices compared to robotic suturing. The laparoscopic needle driver and Da Vinci Si showed a significant shorter median suturing time compared to the FlexDex. This study provides new insight in the further implementation and training of mechanically articulating needle holders to avoid learning curve effects for patients. Future studies should further clarify the potential benefits and limitations of each suturing modality in endoscopic surgery.

## Data Availability

The dataset used during the current study is available from the corresponding author on reasonable request.

## Abbreviations

GOALS: Global operative assessment of laparoscopic skills
NASA-TLX: National aeronautics and space administration task load index
IQR: Interquartile range

## References

[1] Coccolini F (2015). Open versus laparoscopic cholecystectomy in acute cholecystitis. Systematic review and meta-analysis. Int J Surg.

[2] Kasai M (2018). Laparoscopic versus open major hepatectomy: a systematic review and meta-analysis of individual patient data. Surgery.

[3] Bullen NL (2019). Open versus laparoscopic mesh repair of primary unilateral uncomplicated inguinal hernia: a systematic review with meta-analysis and trial sequential analysis. Hernia.

[4] Lacy AM (2002). Laparoscopy-assisted colectomy versus open colectomy for treatment of non-metastatic colon cancer: a randomised trial. Lancet.

[5] Nelson H (2004). A comparison of laparoscopically assisted and open colectomy for colon cancer. N Engl J Med.

[6] Buunen M (2009). Survival after laparoscopic surgery versus open surgery for colon cancer: long-term outcome of a randomised clinical trial. Lancet Oncol.

[7] Lane BR, Campbell SC, Gill IS (2013). 10-year oncologic outcomes after laparoscopic and open partial nephrectomy. J Urol.

[8] Wang YZ (2015). Laparoscopy versus laparotomy for the management of early stage cervical cancer. BMC Cancer.

[9] Lim S (2017). Laparoscopic Suturing as a Barrier to Broader Adoption of Laparoscopic Surgery. Jsls.

[10] Fuchs Weizman N (2015). Survey on barriers to adoption of laparoscopic surgery. J Surg Educ.

[11] Janki S (2017). Ergonomics in the operating room. Surg Endosc.

[12] Sánchez A (2018). Robot-assisted surgery and incisional hernia: a comparative study of ergonomics in a training model. J Robot Surg.

[13] Ruurda JP, van Vroonhoven TJMV, Broeders IAMJ (2002). Robot-assisted surgical systems: a new era in laparoscopic surgery. Ann R Coll Surg Engl.

[14] Boyd WD (2000). A comparison of robot-assisted versus manually constructed endoscopic coronary anastomosis. Ann Thorac Surg.

[15] van der Schatte Olivier RH (2009). Ergonomics, user comfort, and performance in standard and robot-assisted laparoscopic surgery. Surg Endosc.

[16] Stefanidis D (2010). Robotic assistance improves intracorporeal suturing performance and safety in the operating room while decreasing operator workload. Surg Endosc.

[17] Chandra V (2010). A comparison of laparoscopic and robotic assisted suturing performance by experts and novices. Surgery.

[18] Liu R, Liu Q, Wang Z (2021). Worldwide diffusion of robotic approach in general surgery. Updates Surg.

[19] Sieber MA, Fellmann-Fischer B, Mueller M (2017). Performance of Kymerax© precision-drive articulating surgical system compared to conventional laparoscopic instruments in a pelvitrainer model. Surg Endosc.

[20] Di Lorenzo N, Camperchioli I, Gaspari AL (2007). Radius surgical system and conventional laparoscopic instruments in abdominal surgery: application, learning curve and ergonomy. Surg Oncol.

[21] Waseda M (2007). Precision in stitches: Radius Surgical System. Surg Endosc.

[22] Sánchez-Margallo, F., J.A. Sánchez-Margallo, and AmirSzold. Handheld Devices for Laparoscopic Surgery. 2018.

[23] Criss CN (2019). Evaluating a solely mechanical articulating laparoscopic device: a prospective randomized crossover study. J Laparoendosc Adv Surg Tech A.

[24] Leijte E (2020). Robot assisted versus laparoscopic suturing learning curve in a simulated setting. Surg Endosc.

[25] Maniar HS (2005). Comparison of skill training with robotic systems and traditional endoscopy: implications on training and adoption. J Surg Res.

[26] Moorthy K (2004). Bimodal assessment of laparoscopic suturing skills: construct and concurrent validity. Surg Endosc.

[27] Hart S, Hancock PA, Meshkati N (1988). Development of NASA-TLX: results of empirical and theoretical research. Human mental workload.

[28] Frede T (2007). The radius surgical system—a new device for complex minimally invasive procedures in urology?. Eur Urol.

[29] Anderson PL (2016). Comparing a mechanical analogue with the Da Vinci user interface: suturing at challenging angles. IEEE Robot Autom Lett.

[30] Smith CD (2001). Assessing laparoscopic manipulative skills. Am J Surg.

[31] Abiri A (2017). Tensile strength and failure load of sutures for robotic surgery. Surg Endosc.

[32] Cundy TP (2014). Experience related factors compensate for haptic loss in robot-assisted laparoscopic surgery. J Endourol.

[33] Meccariello G (2016). An experimental study about haptic feedback in robotic surgery: may visual feedback substitute tactile feedback?. J Robot Surg.

